# Potentially Avoidable Pediatric Transfers, Tertiary Hospital-Level Care, and Regional Differences in Referral Burden in Greece: A Retrospective Analysis of Transfer Pathways and Costs

**DOI:** 10.3390/children13091171

**Published:** 2026-08-31

**Authors:** Filippos Filippatos, Vasiliki-Maria Kymioni, Dimitra Kallitsounaki, Spyridon Karageorgos, Dimitra-Ifigeneia Matara, Marianna Miliaraki, Stavros Antonopoulos, Konstantinos Kakleas

**Affiliations:** 1 First Department of Pediatrics, Medical School, National and Kapodistrian University of Athens, “Aghia Sophia” Children’s Hospital, 11527 Athens, Greece; filippat@med.uoa.gr (F.F.);; 2 Pediatric Emergency Department, “Aghia Sophia” Children’s Hospital, 11527 Athens, Greece; 3 Second Department of Pediatrics, General Children’s Hospital “Panagiotis and Aglaia Kyriakou”, 11527 Athens, Greece; 4 Pediatric Intensive Care Unit, University Hospital, 71003 Heraklion, Greece

**Keywords:** pediatric transfers, interfacility transport, emergency medical services (EMS), potentially avoidable transfers, healthcare costs, tertiary care referral pathways

## Abstract

**Highlights:**

**What are the main findings?**

- Most pediatric transfers did not meet the predefined criteria for potential avoidability; interfacility transfers accounted for most tertiary hospital-level care/admission events.
- A small proportion of air and sea transfers accounted for nearly all transport expenditure, with marked geographic variation in transport modality and expenditure.

**What are the implications of the main findings?**

- Strengthening primary care, referral coordination, and triage warrants prospective evaluation as a strategy for reducing potentially avoidable transfers.
- High-cost air and sea transfers warrant prospective evaluation of triage, clinical outcomes, and operational expenditure.

**Abstract:**

**Background:** Pediatric transfers to tertiary hospitals may vary according to geographic origin and referral pathways and may impose substantial healthcare costs. However, data regarding pediatric transfer pathways, tertiary hospital-level care/admission, and transport-related expenditure in Greece remain limited. **Methods:** We conducted a retrospective observational study of children aged 0–16 years transferred to a tertiary children’s hospital in Athens, Greece, during 2022–2023. Transfers were classified as prehospital emergency medical service (EMS) transports or interfacility transfers. Post-transfer outcomes were analysed using a predefined binary endpoint: ED/short-stay discharge versus tertiary hospital-level care/admission. Potentially avoidable transfers were identified using conservative predefined resource-utilization criteria. Transport costs were analysed descriptively according to transport modality and geographic region. **Results:** A total of 423 pediatric transfers were analysed, including 216/423 (51.1%) prehospital EMS transports and 207/423 (48.9%) interfacility transfers. Overall, 224/423 children (53.0%) required tertiary hospital-level care/admission, whereas 199/423 (47.0%) were managed as ED/short-stay discharges. Importantly, only 54/199 ED/short-stay discharge cases met the stricter criteria for potential avoidability, representing 54/423 (12.8%) of all transfers. Ground ambulance accounted for most transfers, whereas air and sea transport accounted for only 12.1% of transfers, but 97.0% of transport-related expenditure. In unadjusted pathway-level analysis, interfacility transfer was associated with higher odds of tertiary hospital-level care/admission than prehospital EMS transport (OR 3.72, 95% CI 2.49–5.57; *p* < 0.001) and accounted for 93.8% of total transport costs. **Conclusions:** Most pediatric transfers resulted in tertiary hospital-level care/admission, and only a minority met the predefined criteria for potential avoidability. A substantially higher proportion of interfacility transfers resulted in tertiary hospital-level care/admission, and these transfers accounted for a disproportionate share of recorded transport expenditure.

## 1. Introduction

Regionalized pediatric healthcare systems depend on timely transfer of critically ill or specialized children to tertiary referral centres capable of providing advanced diagnostics, subspecialty consultation, intensive care, and definitive management [1,2,3]. These systems are particularly important in geographically fragmented healthcare settings, where disparities in pediatric expertise and resource availability may substantially influence healthcare utilization and transport-related expenditure [4,5,6].

Greece presents unique challenges for pediatric referral-network organization because of its mainland–island geography, mountainous terrain, seasonal population fluctuations, and unequal regional distribution of pediatric subspecialty services [7,8,9]. Many remote island and rural regions lack continuous access to pediatric intensive care, pediatric surgery, or advanced imaging, frequently necessitating transfer to major referral centres in Athens [10,11].

International studies suggest that a proportion of pediatric interfacility transfers may be avoidable, particularly among children requiring minimal intervention or observation after transfer [12,13,14,15,16]. However, transfer appropriateness represents only one dimension of referral-system performance. Transfer pathways may also be influenced by regional differences in pediatric service availability, referral practices, and access to specialized care, although these factors are not routinely captured in transfer datasets. In geographically dispersed healthcare systems, transport modality and referral-network organization may substantially influence both healthcare utilization and system-level expenditure [17,18,19].

Importantly, prehospital emergency medical service (EMS) transports and interfacility transfers represent fundamentally different healthcare processes. Prehospital EMS transports primarily reflect emergency access patterns and acute illness burden, whereas interfacility transfers more directly reflect referral-network function and tertiary-care dependency [20,21]. Failure to distinguish between these pathways may lead to inaccurate estimation of referral burden and healthcare utilization.

Despite increasing international attention, data regarding pediatric transfer pathways, tertiary hospital-level care/admission, potentially avoidable transfers, and transport-related expenditure in Greece remain limited. Accordingly, the present study aimed to evaluate pediatric transfers to a tertiary children’s hospital in Athens, focusing on transfer pathways, tertiary hospital-level care/admission, geographic referral patterns, potentially avoidable transfers, and associated transport costs within a geographically fragmented healthcare system.

## 2. Methods

### 2.1. Study Design and Setting

This retrospective observational study analyzed all pediatric transfers to Aghia Sophia Children’s Hospital, a tertiary academic children’s hospital in Athens, Greece, and the largest tertiary pediatric referral center in Greece. The study included all children aged 0–16 years who were transferred between 1 December 2022 and 31 August 2023. This study period was predefined because it represented the interval for which complete and internally consistent hospital, transfer, and transport-cost data were available for analysis at the time the study was conducted. No exclusion criteria were applied, regardless of diagnosis, transfer mode, or clinical condition, in order to provide a comprehensive overview of real-world transfer patterns and minimize selection bias, apart from one case with incomplete transfer-source documentation, which prevented classification as either a prehospital EMS transport or an interfacility transfer.

### 2.2. Data Collection and Variables

Data were extracted from the hospital’s electronic medical records and transfer logs and, when necessary, supplemented by National Emergency Medical Service (EKAB) dispatch records to verify transport details. Demographic data for the Greek pediatric population were obtained from the Hellenic Statistical Authority (ELSTAT) (https://www.statistics.gr/en/home/). The following variables were collected for each patient:

- **Demographic characteristics:** age (in years or months for infants) and sex.
- **Clinical information:** primary diagnosis or reason for transfer as documented at hospital arrival. Diagnoses were subsequently grouped into predefined clinically relevant categories according to the principal presenting condition: respiratory, neurologic, gastrointestinal, trauma, surgical, and other. The “other” category comprised low-frequency presentations that did not fit the major diagnostic groups, including minor dermatologic, ophthalmologic, musculoskeletal, genitourinary, and nonspecific complaints. Because individual subcategories had small sample sizes, they were combined into a single category to preserve analytical stability. Classification was performed retrospectively by members of the research team using predefined grouping rules and clinical judgment rather than a formal coding system (e.g., ICD), reflecting real-world documentation practices. Formal independent duplicate classification by more than one investigator was not performed. The same predefined diagnostic classification scheme was used consistently in analyses in which diagnostic category was examined. Representative diagnoses included within each diagnostic category are provided in Supplementary Table S1. This approach may have introduced classification bias, particularly in presentations that could reasonably be assigned to more than one diagnostic category.
- **Transport characteristics:** included mode of transport (ground, air, or sea), geographic origin, and estimated transfer cost. Transport-cost estimates were obtained from hospital financial records and EKAB administrative records. For ground ambulance transfers, the cost assigned to each journey was an estimated administrative amount calculated according to the kilometric distance between the recorded transfer origin and the receiving hospital, using the EKAB administrative costing approach. This amount did not represent a patient-level calculation of actual fuel consumption, personnel time, vehicle depreciation, or other mission-specific expenses, and it should not be interpreted as a reimbursed amount or as the full economic cost of an individual ambulance mission. For air and sea transfers, we used the corresponding administrative cost estimates recorded in available hospital/EKAB records. The economic analysis therefore represents operational transport-cost estimation rather than patient-level or societal costing.
- **Geographic origin:** the broader region from which the child was transferred (Attica/urban, mainland/provincial, or island region). Descriptive analyses used these broad geographic categories, whereas cost analyses additionally incorporated Greek health authority regions to reflect the administrative framework used for transport expenditure recording. For multivariable analyses, geographic origin was collapsed into three categories (Attica, Aegean, and Mainland/Other) to reduce sparse cells and improve model stability.
- **Transfer-source origin:** the immediate site from which transport was initiated (patient’s home or incident scene, hospital, or health centre), distinguishing prehospital EMS transports from interfacility transfers. Because geographic origin and transfer-source origin represent different constructs (location versus referral pathway), both variables were retained in the analyses.
- **Clinical outcomes:** ICU admission upon arrival, length of hospital stay, in-hospital mortality, and final disposition (e.g., discharge, ward admission, or transfer to a surgical or subspecialty service). Standardized baseline illness-severity measures, including triage acuity, Pediatric Early Warning Scores (PEWSs), physiological scores, and validated severity indices, were not consistently available in the retrospective dataset and could not be included in the analyses. All data were de-identified before analysis to ensure patient confidentiality. Trained research personnel collected the data. Transfers were classified a priori into two mutually exclusive categories according to the recorded place of origin. Prehospital EMS transports originated from the patient’s home or incident scene, whereas interfacility transfers originated from another hospital or health centre. Patients were additionally stratified by predefined developmental age groups—infants (<1 year), toddlers (1 to <4 years), preschool children (4 to <6 years), school-age children (6 to <12 years), and adolescents (12–16 years)—and by geographic region (urban, mainland, or island) to facilitate subgroup analyses of referral patterns and associated resource utilization.

### 2.3. Definition of Potentially Avoidable Transfers

For the purposes of this study, potentially avoidable pediatric transfers were operationally defined using a predefined composite endpoint based on post-transfer emergency department (ED) resource utilization rather than ED discharge alone. A transfer was classified as potentially avoidable if all of the following criteria were met: (1) no tertiary hospital-level care/admission following ED evaluation; (2) no intensive care unit (ICU) admission; (3) no advanced diagnostic or therapeutic intervention requiring tertiary hospital-level resources, defined as the absence of invasive procedures or surgery, advanced imaging (CT or MRI), or subspecialty procedural intervention; and (4) no documented escalation of care during ED or short-stay observation beyond routine clinical assessment, basic investigations, monitoring, and supportive treatment. Transfers resulting in ED discharge after advanced diagnostics, specialist evaluation, or extended monitoring were not automatically classified as avoidable, acknowledging that tertiary expertise may still be required even when hospitalization is ultimately unnecessary. Classification was conducted retrospectively by the study investigators through structured review of the complete hospital record for each transferred child using the predefined composite criteria described above. The adjudication incorporated all available post-transfer clinical information, including final disposition, ICU admission, advanced imaging (CT or MRI), invasive procedures or surgery, subspecialty procedural intervention, and documented escalation of care beyond routine assessment, monitoring, basic investigations, and supportive treatment. Because transfer origin and the subsequent clinical course formed part of the medical record, reviewers were not blinded to transfer pathway. Cases were assessed using the predefined criteria. Formal independent duplicate adjudication was not performed, and inter-rater reliability was therefore not assessed. Accordingly, the resulting classification represents a study-specific, resource-based estimate of potential avoidability rather than an independently adjudicated determination of clinical appropriateness. The definition was intentionally conservative to reduce overclassification and align with the previous literature using post-transfer resource utilization as a pragmatic proxy for transfer necessity [14,15]. For descriptive purposes, Supplementary Table S2 compares the remaining transferred children. This comparator was included solely to contextualize the classified potentially avoidable cohort and does not constitute a predefined analytical study group.

### 2.4. Statistical Analysis

Data were analysed using IBM SPSS Statistics Version 25 (IBM Corp., Armonk, NY, USA) [22] and R Version 4.0 (R Foundation for Statistical Computing, Vienna, Austria) [23]. Continuous variables are presented as means with standard deviations or medians with interquartile ranges, as appropriate. Categorical variables are reported as frequencies and percentages. Group comparisons were performed using χ^2^ or Fisher’s exact tests for categorical variables and one-way ANOVA or the Kruskal–Wallis test for continuous variables, as appropriate [24]. Regional study-period transfer rates were compared with those of the Athens/Piraeus reference region using a χ^2^ test for proportions. These rates represent referrals to the study hospital relative to the permanent pediatric population reported by ELSTAT and should not be interpreted as population incidence or total regional transfer activity.

Post-transfer outcome was analysed using a revised binary endpoint: tertiary hospital-level care/admission versus ED/short-stay discharge. ED/short-stay discharge included direct discharge from the emergency department and short-stay observation (≤8 h) without escalation to inpatient, ICU, surgical, or subspecialty-level tertiary care. Tertiary hospital-level care/admission included ward admission, ICU-coded destination, surgical or subspecialty routing, and other non-ED tertiary hospital-level dispositions. This binary endpoint was selected to avoid ambiguity between conventional ward admission and other clinically meaningful tertiary hospital-level dispositions.

Transfer pathway was analysed as the primary explanatory variable. Its association with tertiary hospital-level care/admission was assessed using univariable logistic regression, with prehospital EMS transport as the reference category. Results are reported as unadjusted odds ratios (ORs) with 95% confidence intervals and *p*-values.

Fully adjusted modelling was explored but not retained because sparse data and unstable estimates arose when diagnosis, age, geographic origin, and transport mode were included across several covariate strata. The primary analysis therefore focused on the unadjusted pathway-level association between transfer type and tertiary hospital-level care/admission rather than an independent or causal effect.

Potentially avoidable transfers were defined using the predefined conservative composite endpoint described above and were reported overall and by transfer pathway. ED or short-stay discharge alone was not considered sufficient to classify a transfer as potentially avoidable.

Transfer costs were analysed descriptively because costs were highly right-skewed and driven predominantly by transport modality. Total, mean, median, and proportional costs were calculated by transport mode and health authority region. Sensitivity analyses assessed the robustness of cost concentration under alternative unit-cost assumptions.

Exploratory supplementary regression analyses examined associations of transfer characteristics with transport modality and transport cost, using logistic regression for air/boat versus ground transport and ordinary least-squares regression of log-transformed transport cost, with robust standard errors in the overall cost model; model-specific covariates and reference categories are reported in Tables S3–S5.

Statistical significance was set at *p* < 0.05. Missing data were assessed for all variables. Given the low proportion of missing values, a complete-case analysis was performed for analyses that required those variables.

### 2.5. Ethical Considerations

This study was reviewed and approved by the Ethics Committee of Aghia Sophia Children’s Hospital (Approval Code: 12385/2 June 2023). Given the study’s retrospective, observational nature, informed consent was waived. All procedures adhered to the ethical standards of the Declaration of Helsinki and national guidelines for biomedical research. To protect patient confidentiality, all data were anonymized before analysis, with unique identifiers replaced by coded IDs. Strict data protection protocols were followed throughout data handling and storage. Findings were reported objectively, and all efforts were made to ensure data accuracy, transparency, and integrity.

## 3. Results

### 3.1. Demographic Characteristics

A total of 423 pediatric patients were transferred to the tertiary hospital during the 9-month study period: 94/423 (22.2%) between December and February, 135/423 (31.9%) between March and May, and 194/423 (45.9%) between June and August. The mean age was 7.9 ± 5.3 years (range: 0–16 years), with infants (<1 year) comprising 11.3% of transfers, toddlers (1 to <4 years) 25.2%, preschoolers (4 to <6 years) 11.6%, school-age children (6 to <12 years) 23.1%, and adolescents (12–16 years) 28.8%. Males constituted 52.1% of the cohort. No significant sex difference was observed across age categories (χ^2^ = 2.13, df = 4, *p* = 0.71). Adolescents represented the largest transfer subgroup (28.8%), followed by toddlers aged 1 to <4 years (25.2%). Boys were slightly overrepresented among trauma transfers in the 6–16-year age range (*p* = 0.048).

Of the 423 pediatric transfers analysed, 216/423 (51.1%) were prehospital EMS transports from home or the incident scene and 207/423 (48.9%) were interfacility transfers. The interfacility cohort comprised 183 transfers from another hospital and 24 from a health centre.

**Figure 1:**
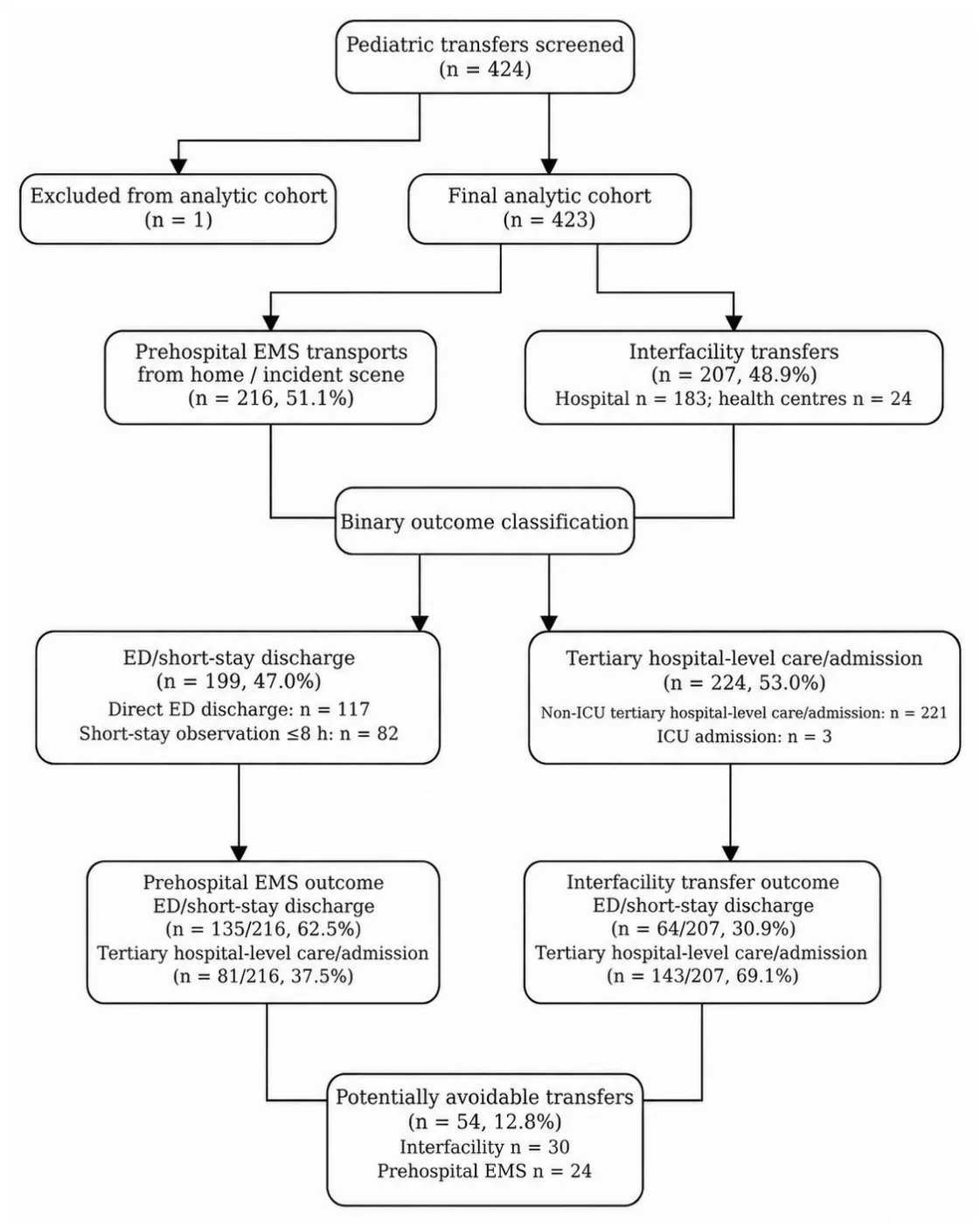
Flow diagram of pediatric transfers included in the study. The final analytic cohort comprised 423 pediatric transfers, classified as prehospital EMS transports (n = 216) or interfacility transfers (n = 207). Post-transfer dispositions included direct ED discharge (n = 117), short-stay observation ≤ 8 h (n = 82), non-ICU tertiary hospital-level care/admission (n = 221), and ICU admission (n = 3). For the primary analysis, direct ED discharge and short-stay observation were combined as ED/short-stay discharge (n = 199), whereas non-ICU tertiary hospital-level care/admission and ICU admission were combined as tertiary hospital-level care/admission (n = 224). Non-ICU tertiary hospital-level care/admission included ward admission, surgical or subspecialty routing, and other non-ED tertiary hospital-level dispositions. Potentially avoidable transfers (n = 54) were identified using the predefined conservative composite resource-utilization criteria. One case was excluded because incomplete transfer-source documentation prevented classification as either a prehospital EMS transport or an interfacility transfer.

Using the predefined binary post-transfer endpoint, 199/423 children (47.0%) were classified as ED/short-stay discharges, while 224/423 (53.0%) required tertiary hospital-level care/admission. This endpoint was used to describe post-transfer resource utilization and should be distinguished from the stricter potentially avoidable-transfer definition. ED/short-stay discharge alone was not considered sufficient to classify a transfer as potentially avoidable. The study flow, transfer pathways, and post-transfer outcome classification are summarized in Figure 1.

Among patients with a documented hospital stay, length of stay did not differ significantly by season. Median (IQR) length of stay was 3.0 days (2.0–5.25) in winter, 3.0 days (2.0–5.0) in spring, and 2.5 days (1.0–6.0) in summer (Kruskal–Wallis *p* = 0.754). Regarding the place of transfer, 216/423 (51.1%) were directly from home, 183/423 (43.3%) from a secondary pediatric hospital, and 24/423 (5.7%) from a peripheral primary health center. Most transported children were from Athens [248/423 (58.6%)], followed by central Greece [66/423 (15.6%)], southern Greece [52/423 (12.3%)], islands [49/423 (11.6%)], and northern Greece [8/423 (1.9%)].

### 3.2. Transfer Origin and Mode of Transport

In total, ground ambulances were the predominant mode of transport (87.9%), followed by air ambulance (9.7%) and boat (2.4%). Transport mode was significantly associated with geographic region (χ^2^ = 89.3, df = 8, *p* < 0.001), with air and sea transfers being more frequent in remote or island areas.

By contrast, interfacility transfers were predominantly conducted by ground ambulance (77.8%), followed by air ambulance (19.3%) and boat ambulance (2.9%), whereas most prehospital EMS transports used ground ambulance (97.7%).

### 3.3. Regional Referral Rates to the Study Hospital and Seasonal Distribution

Observed study-period referral rates to the study hospital differed by geographical region, with the highest rate in southern Greece and the lowest in northern Greece (Table 1). These differences describe referral burden to this center and not regional population incidence. Table 1 summarizes transfers according to broad geographical regions for epidemiological rate estimation, whereas the subsequent economic analyses use Greek health authority divisions. These classification systems are not identical; therefore, transfer counts are not directly comparable across the two tables. For example, Athens/Piraeus is presented as a single geographical category in Table 1, whereas the economic analyses distribute corresponding cases across the Attika–Athens and Piraeus–Aegean administrative regions.

Infants had the highest observed study-period transfer rate from the islands (0.72/1000), nearly twice that observed among infants in Athens (0.39/1000). Children aged 1 to <4 years had a higher transfer rate in Athens (0.72/1000), whereas preschool-aged children from the islands had a comparatively low transfer rate (0.14/1000). These geographic and age-related differences may reflect variation in referral practices, healthcare utilization, population characteristics, or local service availability; however, these potential explanations could not be directly evaluated in the present study.

Transfers peaked in summer (45.9%), compared to spring (31.9%) and winter (22.2%). This seasonal trend was statistically significant (χ^2^ = 27.4, df = 2, *p* < 0.001), and may reflect increased pediatric trauma, tourism, and temperature-related illnesses. Interpretation of regional transfer rates during summer should consider that ELSTAT population estimates reflect permanent residential populations and do not capture temporary increases associated with tourism and seasonal migration. Consequently, seasonal population fluctuations may have influenced study-period transfer-rate estimates across all regions to some extent. However, the effect may have been greater in island and southern regions, where temporary population increases associated with tourism are most pronounced, potentially leading to modest overestimation of transfer rates relative to the true at-risk pediatric population.

### 3.4. Diagnostic Categories and Tertiary Hospital-Level Care/Admission

Respiratory conditions were the most common diagnostic category, followed by neurologic, trauma, gastrointestinal, surgical, and other conditions, according to the predefined diagnostic classification scheme, which was applied consistently across descriptive and inferential analyses (Table 2). Using the binary endpoint, 224/423 children (53.0%) required tertiary hospital-level care/admission, whereas 199/423 (47.0%) were managed as ED/short-stay discharges. The proportion requiring tertiary hospital-level care/admission differed significantly by diagnostic category (χ^2^ = 92.4, df = 5, *p* < 0.001). The highest rates occurred among surgical (90.0%), neurologic (88.9%), and respiratory conditions (70.3%), whereas trauma (44.6%), gastrointestinal conditions (43.8%), and other presentations (10.3%) were more frequently managed as ED/short-stay discharges (Table 2).

When outcomes were examined according to transfer pathway, tertiary hospital-level care/admission occurred in 143/207 interfacility transfers (69.1%) compared with 81/216 prehospital EMS transports (37.5%), indicating that a higher proportion of interfacility transfers resulted in tertiary hospital-level care/admission. Since validated severity measures were unavailable, this difference should not be interpreted as direct evidence of greater baseline illness severity.

Age significantly affected diagnostic distribution (*p* < 0.001). Infants more commonly presented with respiratory disease, whereas adolescents had higher frequencies of trauma and surgical conditions. Sex was not associated with diagnostic pattern (*p* = 0.74). The same predefined diagnostic classification scheme was used consistently in analyses in which diagnostic category was examined.

### 3.5. Potentially Avoidable Transfers

Applying the predefined conservative composite criteria, 54/423 transfers (12.8%) were classified as potentially avoidable. These cases required neither tertiary hospital-level care/admission nor ICU admission, advanced imaging, invasive procedures, surgery, subspecialty procedural intervention, or escalation beyond routine assessment and supportive care. Only 54 of the 199 ED/short-stay discharge cases met this stricter definition; therefore, the 12.8% represents the proportion meeting the study-specific resource-based criteria rather than the prevalence of definitively inappropriate transfers.

Potentially avoidable transfers were most commonly associated with minor trauma (e.g., low-mechanism falls and superficial injuries), uncomplicated gastrointestinal illness (e.g., vomiting or diarrhea without dehydration), mild respiratory symptoms (e.g., uncomplicated upper respiratory infection or mild wheeze), and nonspecific complaints such as fever or abdominal pain without red-flag features. They originated predominantly from urban settings, particularly Athens (78.3%), and were transported almost exclusively by ground ambulance (95%). Adolescents aged 12–16 years represented the largest subgroup (40%). Supplementary Table S2 compares the principal demographic, transport, geographic, and clinical characteristics of the potentially avoidable transfer cohort with the remaining transferred children. Due to the fact that the comparator comprises a heterogeneous population—including children requiring tertiary hospital-level care/admission as well as ED/short-stay discharges that did not satisfy the predefined avoidability criteria—it should not be interpreted as a homogeneous non-avoidable cohort.

Of the 54 potentially avoidable transfers, 30 (55.6%) occurred among interfacility transfers and 24 (44.4%) among prehospital EMS transports, indicating that potentially avoidable transfers arose through both referral pathways, with a slight predominance among interfacility referrals. Since classification was based on retrospective chart review using predefined resource-utilization criteria, these estimates should be interpreted as pragmatic indicators of transfer appropriateness rather than definitive measures of clinical necessity.

### 3.6. Distribution and Determinants of Transfer Costs

The mean transfer cost was €745.56 (SD €2063.87), compared with a median of €15.00, reflecting a markedly right-skewed distribution. The median represents a distance-based administrative estimate—not the actual or reimbursed mission cost—and was determined by the predominance of ground ambulance transfers (372/423, 87.9%), many involving relatively short journeys within metropolitan Athens. In contrast, a small number of high-cost air and sea transfers disproportionately increased the mean and accounted for most overall expenditure.

Cost distribution by transport mode and region, as well as sensitivity analyses, are presented in Table 3, Table 4 and Table 5. Total transport expenditure for the study cohort was €315,370. Cost allocation was overwhelmingly driven by transport modality: air ambulance transfers alone accounted for €280,015 (88.79% of total costs), while boat transfers contributed €26,015 (8.25%). In contrast, ground ambulance transfers, despite representing the vast majority of cases (372/423, 87.9%), accounted for only €9340 (2.96%) of total expenditure. Regional cost estimates for Crete and Macedonia–Thrace were based on only two transfers each and are therefore unstable and should be interpreted descriptively only.

This concentration of costs indicates that recorded operational transport expenditure is determined primarily by a small subset of high-cost transfers rather than by the volume of routine transports. Sensitivity analyses (Table 5) confirmed the robustness of this pattern, demonstrating that even substantial variation in unit costs (±20%) does not materially alter the proportional dominance of air and sea transport. Scenario-specific adjustments affecting only high-cost modalities further amplified this concentration, whereas modifications to ground transport costs had a limited impact on total expenditure despite increasing their proportional share. These findings support interpretation of the economic analysis primarily as a descriptive assessment of cost concentration rather than as evidence of finely resolved patient-level cost determinants.

### 3.7. Structural Drivers of Transfer Costs

Transfer costs demonstrated a highly right-skewed distribution and were driven predominantly by transport modality. Air and sea transfers accounted for a disproportionately large share of total expenditure despite representing a minority of transfers. Supplementary regression analyses explored associations between transfer characteristics and average per-transfer costs; however, these findings should be interpreted cautiously because the economic analyses were primarily descriptive and transport modality accounted for most cost variation. In adjusted models, transfers originating from the Mainland/Other region had approximately 2.4-fold higher geometric mean transport costs than transfers from Attica, whereas estimates for transfers originating from health centres were not reliably estimable because of sparse observations (Tables S3–S5). Although transfers from the Piraeus–Aegean region accounted for most absolute expenditure because of their reliance on air and sea transport, the regression-based geometric mean cost ratios describe average per-transfer costs. Accordingly, the higher Mainland/Other versus Attica cost ratio reflects increased mean per-transfer costs relative to urban transfers and should not be interpreted as inconsistent with the observed concentration of total expenditure in island regions.

### 3.8. Cost Concentration Analysis

**Figure 2:**
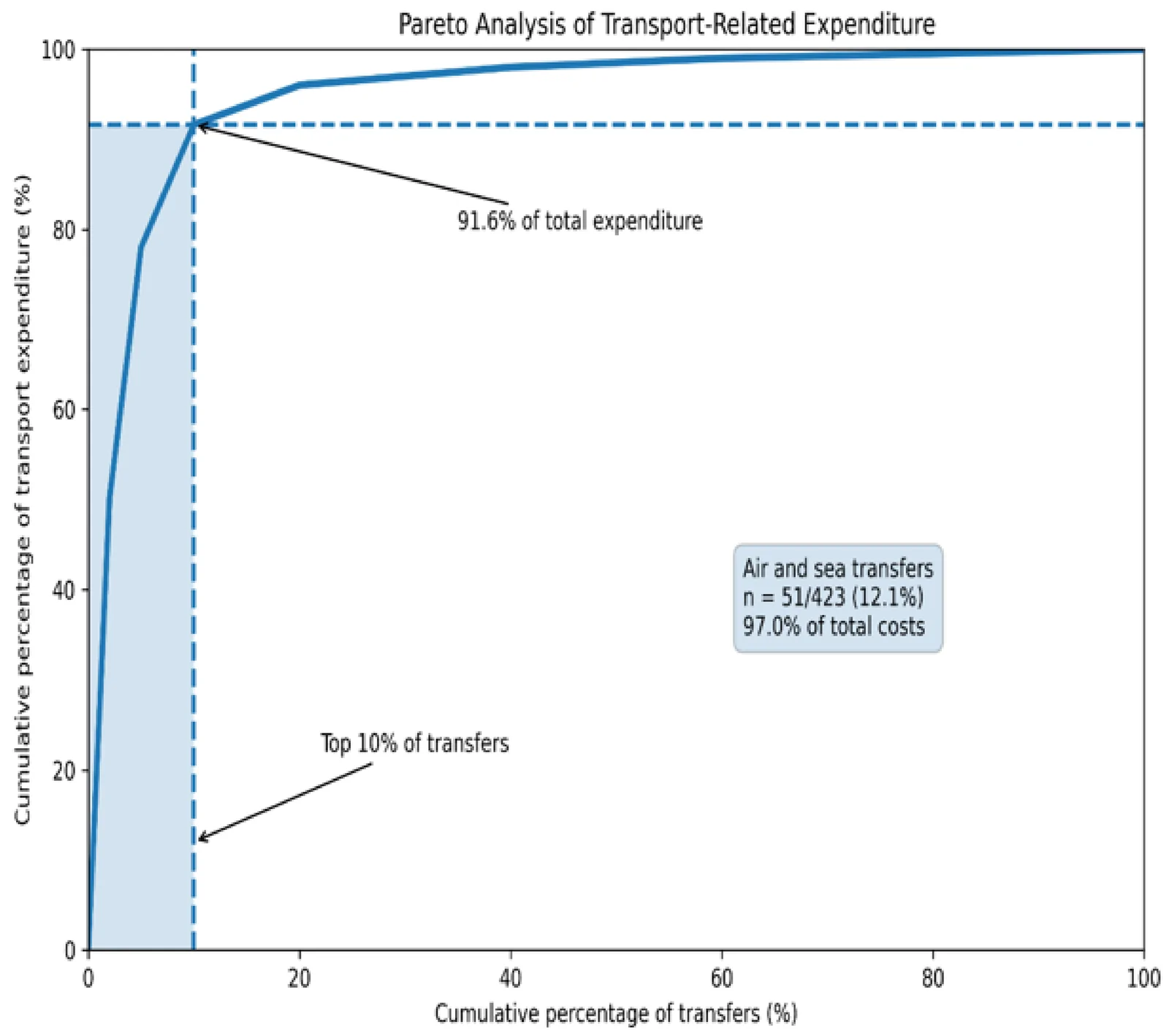
Pareto analysis of transport-related expenditure among pediatric transfers. The highest-cost 10% of transfers accounted for 91.6% of total transport expenditure. Transfers were ranked according to individual transport cost in descending order. The highest-cost 10% corresponded to the 43 most expensive transfers (rounded from 42.3 transfers). Air and sea transfers represented only 12.1% of all transfers (51/423) but accounted for 97.0% of transport-related costs, demonstrating a marked concentration of expenditure among a small subset of transfers.

A Pareto-style cost-concentration analysis demonstrated marked concentration of transport-related expenditure among a small subset of transfers (Figure 2) [25]. Air and sea transfers represented only 51/423 transfers (12.1%) but accounted for 97.0% of total transport expenditure. Furthermore, the highest-cost 10% of transfers accounted for 91.6% of total transport costs, confirming that overall expenditure was driven predominantly by a limited number of high-cost air and sea transports rather than routine ground ambulance transfers.

### 3.9. Association Between Transfer Type and Tertiary Hospital-Level Care/Admission

The primary outcome for revised pathway-level analysis was tertiary hospital-level care/admission versus ED/short-stay discharge. This endpoint was selected to avoid ambiguity between conventional ward admission, short-stay observation, and other non-ED tertiary hospital-level dispositions.

In unadjusted pathway-level analysis, interfacility transfer was associated with higher odds of tertiary hospital-level care/admission than prehospital EMS transport. Tertiary hospital-level care/admission occurred in 143/207 interfacility transfers (69.1%) and 81/216 prehospital EMS transports (37.5%), corresponding to an unadjusted OR of 3.72 (95% CI 2.49–5.57; *p* < 0.001) (Table 6). This estimate should not be interpreted as an independent effect of transfer type.

### 3.10. Interfacility Transfers

Within the interfacility cohort, 183/207 transfers originated from another hospital and 24/207 from a health centre. Using the revised binary outcome, 143/207 interfacility transfers (69.1%) required tertiary hospital-level care/admission, compared with 81/216 prehospital EMS transports (37.5%). Conversely, ED/short-stay discharge occurred in 64/207 interfacility transfers (30.9%) and 135/216 prehospital EMS transports (62.5%).

Potentially avoidable transfers were also reported by transfer type. Of the 54/423 transfers meeting the predefined composite avoidability criteria, 30/207 (14.5%) occurred among interfacility transfers and 24/216 (11.1%) among prehospital EMS transports. Among the 54 potentially avoidable transfers, trauma was the most common diagnostic category (18/54, 33.3%), followed by respiratory conditions (12/54, 22.2%), gastrointestinal conditions (10/54, 18.5%), neurologic conditions (6/54, 11.1%), surgical conditions (5/54, 9.3%), and miscellaneous conditions (3/54, 5.6%).

Transport mode differed markedly by transfer type. Interfacility transfers were conducted by ground ambulance in 161/207 cases (77.8%), air ambulance in 40/207 cases (19.3%), and boat ambulance in 6/207 cases (2.9%). In contrast, prehospital EMS transport was conducted almost exclusively by ground ambulance.

Costs remained highly concentrated in the interfacility cohort, which accounted for €295,850/€315,370 (93.8%) of total transport expenditure. This supports the interpretation that interfacility transfers, although representing approximately half of transfers, accounted for most tertiary hospital-level care/admission events and nearly all transport-related expenditure.

## 4. Discussion

To our knowledge, this is the first Greek study to simultaneously examine transfer appropriateness, tertiary hospital-level care/admission, and transport-related costs within a geographically fragmented pediatric healthcare system. Three key findings emerge. First, most transfers did not meet the predefined criteria for potential avoidability, with only a small proportion (approximately 12.8%) classified as potentially avoidable under a conservative definition based on post-transfer resource utilization. Second, in unadjusted analysis, interfacility transfer was associated with a higher proportion requiring tertiary hospital-level care/admission and accounted for a disproportionate share of recorded transport expenditure. Third, overall expenditure is highly concentrated in a small number of air and sea transfers, indicating that transport modality—rather than transfer volume—was the principal driver of recorded operational transport expenditure.

A central observation is that children are transferred from both nearby and distant regions, including cases that could potentially be managed at lower levels of care. Emergency department discharge alone is not a reliable indicator of unnecessary transfer, as only a subset of cases met strict avoidability criteria. This supports the use of post-transfer resource utilization as a more clinically meaningful proxy for the necessity of transfer [12]. In line with international literature, approximately 10–30% of pediatric transfers—particularly for conditions such as abdominal pain and minor trauma—may be avoidable [12,15]. Avoidability was classified retrospectively using resource-utilization criteria; hence it should be interpreted as a pragmatic system-level indicator rather than a definitive measure of clinical necessity.

Potentially avoidable transfers were predominantly concentrated in urban settings, particularly Athens, and were characterized by limited post-transfer tertiary-resource utilization according to the predefined study criteria. Healthcare access, healthcare-seeking behaviour, system navigation, and caregiver decision-making were not measured and cannot be used to explain this pattern. Previous studies identify these factors as possible determinants of non-urgent ambulance use, but their relevance to our cohort remains uncertain [17,18,19,20]. As shown in Supplementary Table S2, the remaining transfers comprised a clinically heterogeneous population, including both children requiring tertiary hospital-level care/admission and ED/short-stay discharge cases that underwent advanced diagnostics, subspecialty evaluation, procedural intervention, or escalation of care. This supports the use of the predefined composite avoidability definition rather than ED disposition alone as a pragmatic indicator of transfer necessity.

In contrast, transfers from island and remote regions were less frequent but substantially more resource-intensive and appeared to involve children requiring greater tertiary-resource utilization. These findings may be consistent with geographic and organizational differences in referral pathways and local service availability; however, regional healthcare capacity and transfer decision-making were not directly measured. Comparable patterns have been reported in other geographically dispersed healthcare systems, where long-distance transfers are both necessary and resource-intensive [14].

Distinguishing between prehospital EMS transports and interfacility transfers is essential for accurate interpretation because these pathways represent different healthcare processes. Interfacility transfers may be more closely related to referral pathways, regional care capacity, and clinical decision-making in peripheral facilities, whereas prehospital EMS transports—which more often resulted in ED/short-stay discharge—may also be influenced by access barriers and caregiver behaviour. However, these potential determinants were not directly measured, and their contribution to the observed differences remains uncertain. The importance of distinguishing these pathways for evaluating healthcare utilization and system performance has been emphasized in previous research, with similar patterns reported internationally [14,17,18].

Transfer decisions are inherently multifactorial and influenced by clinical condition, local resource availability, transport logistics, and organizational factors that cannot be fully captured retrospectively [14,15,16]. The observed association between transfer pathway and tertiary hospital-level care/admission may therefore be partly explained by differences in diagnosis, age, geographic origin, transport mode, or unmeasured illness severity.

From an operational-cost perspective, recorded transport expenditure was highly concentrated among a small number of air and sea transfers (Figure 2). This finding identifies transport modality and geographic constraints as major drivers of recorded operational expenditure but does not demonstrate inefficiency or cost-effectiveness [14]. Although island transfers accounted for most recorded expenditure because of a small number of costly air and sea evacuations, regression analyses suggested that transfers originating outside Attica had higher average estimated costs than urban transfers.

Because our economic analysis was structured by transport modality and geographic region rather than individual diagnoses, we could not directly quantify diagnosis-specific contributions to transport expenditure. However, respiratory and neurologic conditions had among the highest proportions requiring tertiary hospital-level care/admission and therefore represent plausible major contributors to the burden of interfacility transfers, although this relationship could not be directly evaluated in the present study. Future studies should evaluate diagnosis-specific transport costs within regional pediatric referral networks.

These findings should be interpreted within the broader context of the Greek healthcare system. Previous reports have described structural challenges, including variation in pediatric service availability, primary-care organization, referral pathways and culture, and parental health literacy, which may contribute to observed differences in referral patterns. However, these factors were not directly measured in the present study and should therefore be interpreted as possible explanatory factors rather than direct findings of our analysis [4,21,26,27]. Published national data indicate that healthcare resources and specialist personnel are unevenly distributed across Greece, with greater concentration in major urban centres and lower availability in some island and rural regions [28,29]. These reports suggest that limited local capacity may necessitate transfer of complex pediatric cases, although this mechanism was not directly evaluated in our cohort [30].

International comparisons provide additional context. In the United States, respiratory and neurologic conditions remain leading causes of pediatric admissions, while improvements in outpatient care and coordination have contributed to reductions in hospitalization rates [31,32]. Similarly, studies from North America and Europe report that 10–30% of pediatric interfacility transfers may be avoidable, particularly for low-acuity conditions requiring minimal intervention [12,15,16]. In contrast, healthcare systems with well-developed primary care infrastructure, structured referral pathways, and integrated regional networks—such as those in Northern Europe—tend to demonstrate lower rates of unnecessary transfers and more efficient resource utilization [26]. These systems emphasize gatekeeping, standardized triage, and access to specialist consultation, including telemedicine support [26,27].

These findings derive from a single tertiary referral center and reflect the referral dynamics and institutional context of this setting rather than nationally representative transfer patterns. Nevertheless, because Aghia Sophia Children’s Hospital is the largest tertiary pediatric referral center in Greece and receives transfers from diverse geographic regions, the study provides an internally consistent receiving-center perspective on referral pathways, case complexity, and transport-related resource use.

From a policy perspective, the findings highlight several potential areas for improvement. Strategies proposed in the previous literature include strengthening primary-care access, improving triage, and supporting clinical decision-making; however, the present study did not evaluate these interventions. Evidence from other settings suggests that pediatric observation units can reduce unnecessary admissions and associated costs [33], while standardized referral criteria may improve consistency in transfer decisions. Telemedicine has been proposed in other settings to support clinical decision-making in peripheral facilities [34]; however, its applicability and effectiveness within the Greek pediatric referral system cannot be inferred from the present study.

Based on the patterns observed in our cohort, telehealth-supported triage could be considered for evaluation through two potential pathways: providing real-time specialist input to clinicians in island and remote regions, where high-cost air and sea transport predominates, and supporting telephone- or video-based triage at EMS dispatch or primary/urgent care for urban presentations meeting the potentially avoidable-transfer criteria [35,36,37]. However, this study did not record telemedicine use, and our findings do not demonstrate its effectiveness, transfer prevention, cost reduction, or impact on patient safety. Prospective evaluation within the Greek EKAB and referral network is therefore required before making any policy recommendations.

Previous studies have identified primary-care access and parental health literacy as potential determinants of non-urgent ambulance use [17,19,20,38,39]. These factors were not measured in our cohort, and interventions addressing them require prospective evaluation before policy recommendations can be made. Given the concentration of recorded operational expenditure in air and sea transport, prospective evaluation of triage strategies for high-cost transfers is warranted. Strategies for high-cost transfers should be evaluated prospectively for their effects on transfer appropriateness, patient safety, clinical outcomes, and operational expenditure [14]. Future prospective studies using standardized mission-time definitions could complement these findings by evaluating the operational efficiency and timeliness of pediatric transport pathways.

Because the dataset includes transfers to a single tertiary hospital, regional transfer rates should be interpreted as indicators of referral burden rather than population incidence. Regional healthcare capacity, referral thresholds, transfers to other centers, and temporary or seasonal population movements were not measured. The observed regional differences may therefore have multiple explanations and should not be attributed to any single healthcare-system factor.

## 5. Limitations

This study has several limitations. First, its retrospective observational design limits causal inference and depends on the accuracy and completeness of routinely documented clinical and administrative data. Unmeasured confounding and variability in documentation practices may therefore have influenced classification and outcome assessment. Primary-care access, parental health literacy, regional service availability, referral thresholds, and referral culture were not measured; consequently, their contribution to the observed transfer patterns cannot be determined.

Second, the single-center design limits external validity. Although Aghia Sophia is Greece’s largest tertiary pediatric referral center and receives referrals from multiple regions, the findings reflect its referral catchment, pathways, documentation, and institutional practices and are not nationally representative. Regional rates represent referrals to this hospital rather than total regional transfer activity or population incidence. Regional healthcare capacity, referral thresholds, alternative referral destinations, and temporary population movements were not measured and may have contributed to the observed differences. Moreover, regional cost estimates for Crete and Macedonia–Thrace were based on only two transfers each and are therefore unstable and descriptive only. Multicenter or population-based studies are needed to assess the generalizability of these findings across Greece.

Third, the nine-month study period did not encompass a complete calendar year and therefore excluded the autumn months (September–November). Consequently, the observed seasonal distribution should be interpreted with caution, as inclusion of a complete annual cycle might have altered the relative distribution of transfers across seasons. Future studies including complete calendar-year data are warranted to better characterize annual seasonal variation in pediatric transfer patterns.

Fourth, transport costs reflected administrative operational estimates rather than patient-level micro-costing. Ground ambulance costs were assigned using the EKAB distance-based costing approach between the recorded transfer origin and the receiving hospital; actual fuel consumption, personnel time, vehicle depreciation, return journeys, and other mission-specific resources were not measured. These values should not be interpreted as actual patient-level costs, reimbursement amounts, or societal costs. The analysis therefore characterizes transport-modality-based expenditure and cost concentration but cannot determine precise individual costs, cost-effectiveness, economic efficiency, or the full societal burden of pediatric transport.

Fifth, diagnostic grouping was based on retrospective clinical categorization rather than standardized ICD coding. Although predefined grouping rules were applied consistently, classification required clinical judgment and was not independently duplicated. Misclassification was therefore possible, particularly for overlapping or multisystem presentations, and diagnosis-specific comparisons should be interpreted cautiously.

Sixth, potentially avoidable transfers were classified retrospectively using predefined resource-utilization criteria. Investigators were not blinded to the transfer pathway because transfer origin and the subsequent clinical course formed part of the reviewed record. Independent duplicate adjudication was not performed, and inter-rater agreement could therefore not be assessed. Although the predefined criteria supported consistent classification, reviewer-dependent misclassification and retrospective information bias remain possible. The reported 12.8% should therefore be interpreted as a conservative, exploratory estimate based on study-specific criteria rather than a definitive measure of clinical inappropriateness.

Seventh, validated baseline illness-severity measures, including triage acuity, PEWS, physiological scores, and severity indices, were not consistently available. Baseline severity could therefore not be fully characterized or incorporated into the analyses. Tertiary hospital-level care/admission and resource utilization are post-transfer outcomes rather than validated proxies for initial illness severity. Unmeasured differences in severity may consequently have influenced transfer necessity, resource utilization, and outcome interpretation.

Finally, the OR of 3.72 was derived from a univariable model with transfer type as the sole predictor. Fully adjusted modelling was not retained because sparse data produced unstable estimates across several covariate strata. Residual confounding by diagnosis, age, geographic origin, transport mode, illness severity, and other unmeasured factors therefore remains possible. The OR should be interpreted as an unadjusted pathway-level association rather than an independent or causal effect of transfer type.

## 6. Strengths

To our knowledge, it represents one of the first systematic analyses in Greece to jointly examine potential transfer avoidability, geographic distribution, and recorded operational transport expenditure within a single analytical framework.

A key strength is the comprehensive inclusion of all pediatric transfers over the defined study period, without restriction by diagnosis or transfer modality. This approach minimizes selection bias and provides a real-world representation of transfer practices across diverse pediatric presentations.

The study was conducted at Aghia Sophia Children’s Hospital, the largest tertiary pediatric referral center in Greece, which receives transfers from metropolitan and geographically remote regions. This setting supports a detailed receiving-center assessment of referral pathways, case mix, and resource utilization, while the single-center design limits national generalizability.

Another important strength is the integration of clinical, geographic, and economic data. By linking patient outcomes with transport modality and cost, the study provides a system-level perspective on how geographic origin, referral pathway, and transport logistics are associated with healthcare utilization and expenditure. This integrated approach enables identification of key patterns, including the concentration of costs in a small number of high-resource transfers, which may not be apparent when clinical or economic data are examined in isolation.

The consistent application of the predefined diagnostic grouping scheme in analyses in which diagnostic category was examined supports internal consistency and interpretability, while detailed stratification by age, region, seasonality, and transport mode enables granular subgroup analyses.

Finally, by explicitly distinguishing between prehospital EMS transports and interfacility transfers, the study provides a clearer conceptual framework for evaluating pediatric transfer systems. This distinction allows for a more accurate interpretation of healthcare utilization and highlights different targets for intervention, thereby increasing the study’s relevance for health system planning and policy development.

## 7. Conclusions

Within this single tertiary-centre cohort, pediatric transfers differed according to transfer pathway, geographic origin, requirement for tertiary hospital-level care/admission, and transport modality. Most transferred children required tertiary hospital-level care/admission, whereas only a measurable minority—particularly in urban settings—met predefined criteria for potential avoidability. Interfacility transfers accounted for most tertiary hospital-level care/admission events and transport expenditure, while air and sea transfers accounted for a disproportionate share of recorded operational transport expenditure despite representing a minority of cases.

These findings provide insight into referral patterns and resource utilization within a tertiary referral-centre setting. Regional service organization, referral coordination, primary-care access, and telemedicine-supported triage represent plausible explanatory factors or potential interventions rather than direct findings of this study. Their effects on transfer patterns, operational expenditure, patient safety, and clinical outcomes remain uncertain and require prospective multicentre evaluation incorporating standardized severity assessment. Such studies could inform evidence-based strategies for optimizing pediatric transfer networks.

## Figures and Tables

**Table 1:** Observed pediatric referral rates to the study hospital during the nine-month study period, by broad geographical region (per 1000 pediatric population) (according to ELSTAT population data).

Geographical Region	Population	Transfers	Rate per 1000	*p*-Value (vs. Athens) *
Athens/Piraeus	578,165	248	0.43	Reference
Islands	121,785	49	0.40	0.739
South Greece	79,238	52	0.66	0.0065
North Greece	453,369	8	0.02	<0.001
Central Greece	397,661	66	0.17	<0.001

* *p*-values represent comparisons of study-period transfer rates with Athens/Piraeus using χ^2^ tests for proportions. Footnotes: Regions are grouped according to broad geographical areas for epidemiological rate estimation using ELSTAT population data. These geographical categories differ from the Greek Health Authority regions used for the subsequent economic analyses; therefore, transfer counts are not directly comparable between the two tables. Rates were calculated using permanent pediatric population estimates from ELSTAT. They represent referrals to the study hospital and do not account for transfers to other centers, regional healthcare capacity, referral thresholds, or temporary population movements.

**Table 2:** Diagnosis Distribution According to Post-Transfer Outcome.

Diagnosis	Tertiary Hospital-Level Care/Admission n/N (%)	ED/Short-Stay Discharge n/N (%)
Respiratory	97/138 (70.3)	41/138 (29.7)
Neurologic	40/45 (88.9)	5/45 (11.1)
Gastrointestinal	21/48 (43.8)	27/48 (56.3)
Trauma	29/65 (44.6)	36/65 (55.4)
Surgical	27/30 (90.0)	3/30 (10.0)
Other	10/97 (10.3)	87/97 (89.7)
Total	224/423 (53.0)	199/423 (47.0)

**Table 3:** Distribution of Transfer Costs by Transport Mode.

Mode	Transfers (n)	Total Cost (€)	Mean Cost (€)	Median Cost (€)	% of Total Budget
Air ambulance	41	280,015	6829.63	7000.00	88.79
Boat ambulance	10	26,015	2601.50	3000.00	8.25
Ground ambulance	372	9340	25.11	15.00	2.96
Total	423	315,370	745.56	15.00	100

Footnote: Costs represent administrative operational estimates rather than patient-level micro-costs or reimbursement amounts. Ground ambulance costs were assigned according to the kilometric distance between the recorded transfer origin and the receiving hospital using the EKAB administrative costing approach. The overall median of €15 reflects the predominance of ground ambulance transfers, particularly relatively short-distance transfers within the Athens metropolitan area.

**Table 4:** Total cost distribution by Greek Health Authority Region.

Region Code (Health Authority)	Transfers (n)	Total Cost (€)	Mean (€)	Median (€)	% of Total Budget
Piraeus–Aegean Islands	127	279,430	2200.24	15.00	88.60
Epirus–Ionian Islands–Peloponnese	85	17,655	207.71	45.00	5.60
Crete	2	14,000	7000.00	7000.00	4.44
Attika–Athens	164	2460	15.00	15.00	0.78
Thessaly–Sterea	43	1795	41.74	45.00	0.57
Macedonia–Thrace	2	30	15.00	15.00	0.01
Total	423	315,370	745.56	15.00	100

Footnote: Regions are grouped according to the administrative divisions of the Greek Health Authorities used for transport expenditure recording. These administrative regions differ from the broader geographical regions used for epidemiological rate estimation in Table 1; therefore, transfer counts are not directly comparable between the two tables. Crete and Macedonia–Thrace included only two transfers each; their mean and median cost estimates are unstable and should not be interpreted as reliable regional estimates.

**Table 5:** Sensitivity analysis on the mode of transfer/costs.

Scenario	Total Cost (€)	Air Share (%)	Boat Share (%)	Ground Share (%)
Baseline (observed)	315,370	88.79	8.25	2.96
All unit costs +20%	378,444	88.79	8.25	2.96
All unit costs −20%	252,296	88.79	8.25	2.96
Air/sea +20% only	376,576	89.23	8.29	2.48
Air/sea −20% only	254,164	88.13	8.19	3.68
Ground unit cost doubled (≈€30 avg)	324,710	86.23	8.01	5.76
Ground overridden to €60 each	328,350	85.26	7.92	6.81

**Table 6:** Association Between Transfer Type and Tertiary Hospital-Level Care/Admission.

Transfer Type	Tertiary Hospital-Level Care/Admission	ED/Short-Stay Discharge	Unadjusted OR	95% CI	*p*
Prehospital EMS	81/216 (37.5%)	135/216 (62.5%)	1.00 reference	—	—
Interfacility transfer	143/207 (69.1%)	64/207 (30.9%)	3.72	2.49–5.57	<0.001

Footnote: The odds ratio was derived from a univariable logistic regression model with transfer type as the sole predictor. It represents an unadjusted pathway-level association and not an independent or causal effect.

## Data Availability

The raw data supporting the conclusions of this article will be made available by the authors on request.

## Supplementary Materials

The following supporting information can be downloaded at: https://www.mdpi.com/article/10.3390/children13091171/s1, Table S1: Representative diagnoses included in each diagnostic category; Table S2: Principal demographic, transport, geographic, and clinical characteristics of potentially avoidable transfers compared with the remaining transferred children; Table S3: Two-part model, Part 1 (logistic regression): predictors of air/boat transport; Table S4: Two-part model, Part 2 (ordinary least-squares regression on log-transformed cost): cost among air/boat transfers; Table S5: Overall ordinary least-squares regression of log-transformed transport cost using robust standard errors.
