# Supplementary material for: Potentially Avoidable Pediatric Transfers, Tertiary Hospital-Level Care, and Regional Differences in Referral Burden in Greece: A Retrospective Analysis of Transfer Pathways and Costs
Source: Children (Basel). 2026 Aug 31;13(9):1171. doi: 10.3390/children13091171 (PMC13605878; doi:10.3390/children13091171)
Supplement: Supplementary file 1 [file children-13-01171-s001.zip › children-4481810-supplementary.pdf]

Supplementary Table S1- Representative diagnoses included in each diagnostic category.

Category	Representative diagnoses
**Respiratory**	**Bronchiolitis, pneumonia, asthma, URTI**
**Neurologic**	**Seizures, febrile seizure, headache**
**GI**	**Gastroenteritis, vomiting, abdominal pain**
**Trauma**	**Falls, head injury, fractures**
**Surgical**	**Appendicitis, testicular torsion**
**Other**	**Dermatologic, ophthalmologic, GU, intoxications**

Abbreviations: GI, gastrointestinal; GU, genitourinary; URTI, upper respiratory tract infection.

Supplementary Table S2- Principal demographic, transport, geographic, and clinical characteristics of potentially avoidable transfers compared with the remaining transferred children.

Characteristic	Potentially avoidable transfers (n=54)	Remaining transferred children (n=369)
**Total**	54 (12.8%)	369 (87.2%)
**Interfacility transfers**	30 (55.6%)	177 (48.0%)
**Prehospital EMS transports**	24 (44.4%)	192 (52.0%)
**Predominant geographic origin**	Urban (Athens) 78.3%	Mixed urban, mainland and island referrals
**Predominant transport mode**	Ground ambulance (95%)	Ground ambulance remained the predominant transport mode, although this group included almost all air and sea transfers
**Largest age group**	Adolescents (40%)	All pediatric age groups represented; children requiring tertiary hospital-level care/admission occurred across all age groups.
**Typical presentations**	Minor trauma, mild respiratory illness, uncomplicated gastrointestinal illness, nonspecific fever/abdominal pain	Respiratory illnesses requiring admission, neurologic disorders (particularly seizures), surgical conditions (appendicitis, testicular torsion), gastrointestinal disease requiring tertiary hospital-level care/admission, trauma requiring hospital admission, and other conditions requiring tertiary hospital-level evaluation, advanced imaging, surgery, subspecialty intervention, or escalation of care

The remaining transfers comprise a heterogeneous comparator group including (i) children requiring tertiary hospital-level care/admission (n = 224) and (ii) ED/short-stay discharge cases (n = 145) that did not fulfil the predefined composite criteria for potential avoidability, because they underwent advanced imaging, specialist evaluation, invasive procedures, surgery, subspecialty procedural intervention, or escalation of care. Consequently, this comparator should not be interpreted as a homogeneous "non-avoidable" cohort.

Supplementary Table S3 — Two-part model, Part 1 (logistic regression): predictors of air/boat transport.

Predictor	OR	95% CI	p
Age	0.93	0.86–1.02	0.129
Male	0.85	0.37–1.98	0.710
Aegean	57.64	6.79–489.25	<0.001
Mainland/Other	0.98	0.09–10.21	0.986
Hospital origin	15.34	3.22–72.96	0.001
*Health Center origin	—	—	—

Footnote: Outcome: air/boat versus ground ambulance. Reference categories were female sex, Attica, and patient/home or incident-scene origin. OR, odds ratio; CI, confidence interval.
*Health-centre origin was not reliably estimable because of sparse-data instability and low event counts within selected covariate strata.

Supplementary Table S4— Two-part model, Part 2 (ordinary least-squares regression on log-transformed cost): cost among air/boat transfers.

Predictor	Cost ratio*	95% CI	p
Age (per year)	1.00	0.99–1.00	0.386
**Boat vs air**	**0.41**	0.36–0.46	**<0.001**
Mainland/Other vs Aegean	1.00	0.96–1.03	0.862

*Cost ratio = exp(β) from the log-cost model. Reference categories were air transport and Aegean geographic origin. CI, confidence interval.

Supplementary Table S5. — Overall ordinary least-squares regression of log-transformed transport cost using robust standard errors.

Predictor	Geometric mean ratio	95% CI	p
Age (per year)	1.0	0.99–1.01	0.263
Male (vs female)	1.0	0.9–1.2	0.986
Aegean (vs Attica)	1.2	1.0–1.5	0.051
**Mainland/Other (vs Attica)**	**2.4**	2.1–2.8	**<0.001**
**Air vs ground**	**312.7**	204.1–479.0	**<0.001**
**Boat vs ground**	**95.7**	31.2–293.1	**<0.001**

Footnote: Outcome: log-transformed transport cost. Reference categories were female sex, Attica, and ground ambulance. Effect estimates are presented as geometric mean ratios [exp(β)]. CI, confidence interval; SE, standard error.
